# A *C. trachomatis* Cloning Vector and the Generation of *C. trachomatis* Strains Expressing Fluorescent Proteins under the Control of a *C. trachomatis* Promoter

**DOI:** 10.1371/journal.pone.0057090

**Published:** 2013-02-18

**Authors:** Hervé Agaisse, Isabelle Derré

**Affiliations:** Department of Microbial Pathogenesis, Yale University School of Medicine, New Haven, Connecticut, United States of America; University of Würzburg, Germany

## Abstract

Here we describe a versatile cloning vector for conducting genetic experiments in *C. trachomatis*. We successfully expressed various fluorescent proteins (i.e. GFP, mCherry and CFP) from *C. trachomatis* regulatory elements (i.e. the promoter and terminator of the *incDEFG* operon) and showed that the transformed strains produced wild type amounts of infectious particles and recapitulated major features of the *C. trachomatis* developmental cycle. *C. trachomatis* strains expressing fluorescent proteins are valuable tools for studying the *C. trachomatis* developmental cycle. For instance, we show the feasibility of investigating the dynamics of inclusion fusion and interaction with host proteins and organelles by time-lapse video microscopy.

## Introduction

*Chlamydia* species are obligate intracellular Gram-negative bacterial pathogens that infect genital, ocular and pulmonary epithelial surfaces. *Chlamydia* are characterized by a biphasic developmental cycle that occurs exclusively in the host cell. The bacteria alternate between an infectious form called the elementary body (EB) that is characterized by a condensed nucleoid, and an intracellular replicative form termed the reticulate body (RB). Once internalized, *Chlamydia* resides in a membrane bound compartment, named the inclusion. Shortly after uptake, an uncharacterized switch occurs leading to the differentiation of EBs into RBs. The RBs then start to replicate until the inclusion occupies a large part of the cytosol of the host cell. Midway through, the developmental cycle becomes asynchronous and RBs start to differentiate back into EBs. At the end of the cycle, which last two to three days depending on the species, EBs are released from the host cell allowing infection of neighboring cells [1], [2].

To establish and maintain their intracellular niche, *Chlamydia* release effectors proteins into the host cell cytosol. Some target cellular organelles or signaling pathways, while others act directly at the inclusion membrane [3]–[5]. Once internalized [6]–[8], *Chlamydia* directs the trafficking of the nascent inclusion to a perinuclear localization *via* a mechanism involving microfilaments, microtubules and the motor protein dynein [9]. The inclusion does not interact with the endocytic pathway [9], [10] and is encased in a scaffold of host actin and intermediate filaments that maintain vacuole integrity [11]. Infection induces Golgi fragmentation and formation of Golgi ministacks that surround the inclusion to favor the interception of exocytic vesicles and lipids, such as cholesterol and sphingomyelin [12], [13]. Membrane contact sites between *C. trachomatis* inclusion membrane and endoplasmic reticulum patches have also been described [14]. Localization of the ceramide transfer protein, CERT, and the sphingomyelin synthase 2, SMS2, at these points of contact may be involved in sphingomyelin synthesis directly at the inclusion membrane, representing an alternative route for lipids acquisition [14], [15].

Genetic intractability of obligate intracellular pathogens has made it challenging to fully dissect the role of virulence factors involved in pathogenesis. Over the past 10 years, significant advances have occured and elaborated genetic systems have been developed for most obligate intracellular pathogens [16].

To date *Coxiella burnetii*, the causative agent of Q fever [17], has the most comprehensive set of genetics tools including i) transposons used to generate mCherry expressing *Coxiella* strains, random transposition mutagenesis [18] and site-specific transposition integration [19], ii) shuttle vectors allowing mutant complementation and ß-lactamase- or CyA-based secretion assay of type IV effectors [19], [20], iii) an anhydrotetracycline-inducible system [19] and iv) two systems for targeted gene deletion [21]. The development of a medium that supports axenic growth of *Coxiella* [22] has certainly facilitated the development of *Coxiella* genetic tools, but major advances have also been accomplished for true obligates such as *Rickettesiae* spp. Allelic exchange of the phospholipase D gene of *R. prowazekii* has been reported [23], but genetic manipulation of several *Rickettesiae* species has mostly relied on random transposition generating mCherry and GFP expressing strains [24]–[27], and most importantly in the context of the study of host-pathogen interaction, an insertional inactivation mutant strain that is defective for actin-based motility [28].

Genetic tools to study *Chlamydia* pathogenesis have also emerged. In 1998, the sequence of the *C. trachomatis* genome revealed the presence of DNA repair and recombination systems, indicating that *C. trachomatis* was capable of recombination [29]. Several studies have since provided evidence that lateral gene transfer between *C. trachomatis* strains, or different *Chlamydia* species, occurred *in vitro* and *in vivo* [30]–[34].

The first observation of *C. trachomatis* transformation by electroporation leading to transient expression of chloramphenicol resistance was reported in 1994 [35] and in 2009, allelic exchange using circular and linear DNA was reported in *C. psittaci* [36]. Over the past year major advances have occurred and *C. trachomatis* is now considered an obligate intracellular pathogen for which genetic manipulation is still challenging but certainly not impossible. A transformation system, based on calcium rather than electroporation, has been developed and *E. coli-C. trachomatis* shuttle plasmids were successfully introduced and maintained in *C. trachomatis* leading to *C. trachomatis* strains that were resistant to ß-lactams and expressed GFP [37]. Transposition mutagenesis is yet to be developed, but chemical mutagenesis has been used to generate targeted *C. trachomatis* mutant in the tryptophan synthesis pathway [38]. In combination with genome sequencing and a system of DNA exchange among *Chlamydia* strains, a collection of mutants with distinct phenotypes, including mutants with altered glycogen metabolism and with disrupted type II secretion, were also generated [39].

In an effort to further develop *C. trachomatis* genetic tools, that could lead to routine mutation and complementation, we have developed a versatile cloning vector for *C. trachomatis*. We successfully expressed various fluorescent proteins (i.e. GFP, mCherry and CFP) from the *C. trachomatis incDEFG* operon promoter and showed that the transformed strains produced wild type amounts of infectious particles and recapitulated major features of the *C. trachomatis* developmental cycle. *C. trachomatis* strains expressing fluorescent proteins are valuable tools for the study of the *C. trachomatis* developmental cycle by time lapse video microscopy, but more importantly, our cloning vector will be a valuable tool for mutant complementation as well as expression of wild type, mutated or tagged *C. trachomatis* proteins to investigate their role during the *C. trachomatis* developmental cycle.

## Materials and Methods

### Ethics statement

All genetic manipulations and containment work were approved by the Yale Biological Committed and are in compliance with the section III-D-1-a of the National Institutes of Health guidelines for research involving recombinant DNA molecules.

### Cell lines and bacterial strains

HeLa cells cells were obtained from ATCC (CCL-2) and cultured at 37°C with 5% CO_2_ in DMEM high glucose (Invitrogen) supplemented with 10% heat inactivated FBS (Invitrogen). *C. trachomatis Lymphogranuloma venereum, Type II* were obtained from ATCC (L2/434/Bu VR-902B). *Chlamydia* propagation and infection was performed as previously described [40].

### Plasmid construction

Restriction enzymes and T4 DNA ligase were obtained from New England Biolabs (Ipswich, MA). PCR was performed using Herculase DNA polymerase (Stratagene). PCR primers were obtained from Integrated DNA Technologies and their sequence is listed in Table S1. The maps of the plasmids described below were designed using Vector NTI (Invitrogen).

### Construction of p2TK2

p2TK2 was amplified from pGEX-2TK (GE Healthcare) with Mod2TK2-5-Nde and Mod2TK2-3-Nde primers. The corresponding PCR product, containing the *E. coli* origin of replication, the ß-lactamase gene and a multiple cloning site, was circularized after *Nde*I digest.

### Construction of p2TK2-SW2

pSW2 was obtained by *BamH*I digest of pGFP: SW2 [37] and gel purification of the ∼7 kb band corresponding to pSW2. To construct p2TK-SW2, *BamH*I digested pSW2 was cloned into the *BamH*I site of p2TK2.

### Construction of p2TK2-SW2 IncDProm-RSGFP-IncDTerm

DNA fragments corresponding to the intergenic region upstream (IncD Prom) and downstream (IncD Term) of the *incDEFG* operon were amplified by PCR from *C. trachomatis* genomic DNA using primers IncDProm&Orf-5-Kpn and RSGFP-START-3 (IncD Prom) and RSGFP-STOP-5 and IncDTerm-3-Not (IncD Term), respectively. A DNA fragment corresponding to RSGFP was amplified from pGFP: SW2 using primers RSGFP-START-5 and RSGFP-STOP-3. A DNA fragment corresponding to IncDProm-RSGFP-IncDTerm was then amplified by overlapping PCR and cloned into the *Kpn*I/*Not*I sites of p2TK2. p2TK2--SW2 IncDProm-RSGFP-IncDTerm was obtained by cloning the pSW2 plasmid into the *BamH*I site of p2TK2 IncDProm-RSGFP-IncDTerm. Please note the opposite orientation of pSW2 in p2TK2--SW2 IncDProm-RSGFP-IncDTerm and p2TK2-SW2.

### Construction of p2TK2-SW2 IncDProm-mCherry-IncDTerm

DNA fragments corresponding to the intergenic region upstream (IncD Prom) and downstream (IncD Term) of the *incDEFG* operon were amplified by PCR from *C. trachomatis* genomic DNA using primers IncDProm&Orf-5-Kpn and mCherry-START-3 (IncD Prom) and mCherry-STOP-5 and IncDTerm-3-Not (IncD Term), respectively. A DNA fragment corresponding to mCherry was amplified using primers mCherry-START-5 and mCherry-STOP-3. A DNA fragment corresponding to IncDProm-mCherry-IncDTerm was then amplified by overlapping PCR and cloned into the *Kpn*I/*Not*I sites of p2TK2-SW2.

### Construction of p2TK2-SW2 IncDProm-CFP-IncDTerm

DNA fragments corresponding to the intergenic region upstream (IncD Prom) and downstream (IncD Term) of the *incDEFG* operon were amplified by PCR from *C. trachomatis* genomic DNA using primers IncDProm&Orf-5-Kpn and CFP-START-3 (IncD Prom) and CFP-STOP-5 and IncDTerm-3-Not (IncD Term), respectively. A DNA fragment corresponding to CFP was amplified using primers CFP-START-5 and CFP-STOP-3. A DNA fragment corresponding to IncDProm-CFP-IncDTerm was then amplified by overlapping PCR and cloned into the *Kpn*I/*Not*I sites of p2TK2-SW2.

### *C. trachomatis* transformation

The following protocol was adapted from Wang *et. al.* [37]. HeLa cells and *C.trachomatis* L2 were obtained from ATCC. The transformed plasmids were extracted from the *E. coli* GM2163 (*dam* ^−^ *dcm* ^−^) strain using an endofree plasmid maxi Kit (Qiagen). Infected cells were cultured in DMEM High Glucose supplemented with 10% heat inactivated FBS (Invitrogen). Penicillin G and Cycloheximide were from Sigma. The optimal penicillin concentration to select the transformants (1 U/ml) was empirically determined by serial dilution. Once established, the transformed strains were cultured in the presence of 10 U/ml of penicillin.

For one transformation:

**Day 1.** *C. trachomatis* L2 in 10 µl of SPG, 6 µg of plasmid DNA in 10 µl of water and 200 µl of CaCl_2_ Buffer (10 mM Tris, 50 mM CaCl_2_ pH 7.4) were incubate for 30 minutes at room temperature. 4×10^6^ trypsinized HeLa cells were pelleted, washed once with PBS and resuspended in 200 µl of CaCl_2_ Buffer. After 30 minutes, HeLa cells (200 µl) were added to the *C. trachomatis* L2/DNA mix and incubated for an additional 20 minutes at room temperature with mixing by pipetting up and down every 5 minutes. At the end of the 20 minutes, 100 µl of *C. trachomatis* L2/plasmid DNA/HeLa mix was added to a 6 well containing 3 ml of media and incubated for 48 h at 37°C in the presence of 5% CO_2_. The amount of *C. trachomatis* to be used was empirically determined so that 100% of the cells were infected.

**Day 2.** 4×10^6^ HeLa cells were seeded in a 10 cm^2^ dish.

**Day 3.** 100% of the cells (from day 1) were infected and displayed wild type inclusions. The media was removed, the infected cells were lyzed by addition of 2 ml of water, scrapped, collected and spun for 5 minutes at 1,200 rpm. The supernatant was diluted in 10 ml of media containing 1 U/ml penicillin and 1 µg/ml Cycloheximide. The dilution was empirically determine so that when added to the cell monolayer seeded on day 2 and incubated for 48 h at 37°C in the presence of 5% CO_2_, 100% of the cells would be infected.

**Day 4.** 4×10^6^ HeLa cells were seeded in a 10 cm^2^ dish.

**Day 5.** 100% of the cells from day 3 were infected. The inclusions were large but contained aberrant bodies. The media was removed, the infected cells were lyzed (by scrapping the cells in 2 ml of water and passing through a 26 G1/2 needle 5 times), collected and spun for 5 min at 1,200 rpm. The 2 ml of lysate was diluted in 8 ml of media containing 1 U/ml penicillin and 1 µg/ml Cycloheximide, transferred to the monolayer seeded on day 4 and incubated for 3 days at 37°C in the presence of 5% CO_2_.

**Day 7.** 4×10^6^ HeLa cells were seeded in a 10 cm^2^ dish.

**Day 8.** Some wild type inclusions were observed and the amplification process started. The media was removed, the infected cells were lyzed (by scrapping the cells in 2 ml of water and passing through a 26 G1/2 needle 5 times), collected and spun for 5 min at 1,200 rpm. The 2 ml of lysate was diluted in 8 ml of media containing 10 U/ml penicillin and 1 µg/ml Cycloheximide, transferred to the monolayer seeded on day 7 and incubated for 2–3 days at 37°C in the presence of 5% CO_2_.

The amplification process was repeated until enough infectious particles were recovered to generate a frozen stock and proceed to purification.

### Plasmid and genomic DNA extraction

Confluent HeLa cells in 10 cm2 dishes were infected with the parental or the transformed strains at an MOI of 1 and the bacteria were harvested 48 h post infection. Plasmid and total (plasmid and genomic) DNA were respectively prepared using a QIAprep Spin Miniprep Kit (Qiagen) and an illustra bacteria genomicPrep Mini Spin Kit (GE Healthcare), according the manufacturer recommendations.

### Verification of the integrity of the *incDEFG* locus by PCR

*C. trachomatis* total (plasmid and genomic) DNA was used a template. The ChrUpIncDProm and ChrDwnIncDTerm primers were used to amplify the *incDEFG* operon from the chromosome. The DNA fragment corresponding to the *rsgfp* gene, flanked by the *incD* promoter and terminator, was amplified using the primers PlasmUpIncDProm and PlasmDwnIncDTerm. The DNA fragments corresponding to the *mcherry* or *cfp* gene, flanked by the *incD* promoter and terminator, were amplified using the primers 2TK2Fwd and SW2Rev2.

### Southern Blot

*C. trachomatis* total (plasmid and genomic) DNA was digested with *BamH*I, separated by agarose gel electrophoresis and transferred to positively charged nylon membrane. Hybridization with digoxigenin-11-dUTP-labeled probes was performed overnight at 50°C and DIG detection was performed according to the manufacturer recommendation (Roche). The 2TK2 probe was a 2,021 bp DNA fragment corresponding to the p2TK2 plasmid and was generated using the primers Mod2TK2-5-Nde and Mod2TK2-3-Nde. The SW2 probe was a 500 bp DNA fragment corresponding to pSW2 plasmid and was generated using the primers SW2ProbeFwd and SW2ProbeRev.

### Immunofluorescence and microscopy

At the indicated times, the cells seeded onto glass coverslips were fixed for 30 min in PBS containing 4% paraformaldehyde. Immunostaining was performed at room temperature. Antibodies were diluted in PBS containing 0.1% BSA and 0.1% Triton X-100. Samples were washed with PBS and examined under an epifluorescence or spinning disc confocal microscope.

### Infectious progeny production

HeLa cells were collected at the indicated time post infection, lyzed with glass beads and dilutions of the lysate were used to infect fresh HeLa cells. The cells were fixed 24 h post infection and the number of inclusion forming units (IFUs) was determined after assessment of the number of infected cells by immonulabelling.

### Antibodies

The following primary antibodies were used: goat polyclonal anti-MOMP (1∶300, Virostat), rabbit polyclonal anti-*C. trachomatis* IncA (1∶200, kindly provided by T. Hackstadt, Rocky Mountain Laboratories), chicken polyclonal anti-CERT (1∶200, Sigma), mouse monoclonal anti-GM130 (1∶300, BD Biosciences) and mouse monoclonal anti-Vimentin (1∶500, Sigma).

The following secondary antibodies were used: donkey anti-goat AlexaFluor 488 antibody (1∶1,000, Molecular Probes), goat anti-rabbit AlexaFluor 594 or 488 antibody (1∶1,000, Molecular Probes), FITC or TRICT donkey anti-chicken IgY antibody (1∶500, Jackson ImmunoResearch) and goat anti-mouse AlexaFluor 594 or 488 antibody (1∶1,000, Molecular Probes).

### DNA transfection

DNA transfection was performed using Fugene 6 according to the manufacturer recommendations.

### Time-lapse video microscopy

HeLa cells were seeded on 35-mm imaging dishes (MatTek, Ashland, MA) and transfected with the indicated construct 18 hrs prior to infection with the indicated fluorescent *C. trachomatis* strain. At the indicated times post infection, images were captured every 20–30 min on a Nikon TE2000E spinning disc confocal microscope equipped with a humidified live cell environmental chamber set at 37°C and 5% CO_2_. The Volocity software (Improvision, Lexington, MA) was used to analyze and process the data. Videos were saved in QuickTime format using Sorenso 3 compression.

## Results and Discussion

### Generation of a versatile cloning vector for *C. trachomatis*

**Figure 1:**
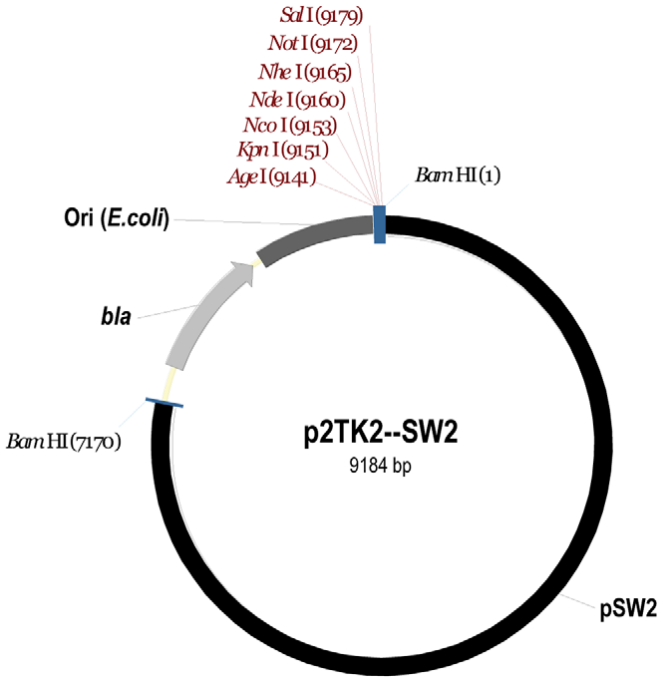
A versatile cloning vector for ***C. trachomatis***. Map of p2TK-SW2. The pSW2 plasmid is shown in black, the *E.coli* origin of replication in dark grey and the ampicillin resistance cassette (*bla*) in light grey. Unique restriction sites of the multiple cloning site are shown in red.

With the objective of developing a versatile cloning vector for *C. trachomatis*, we first generated p2TK2, a minimal plasmid for replication in *E. coli*, containing an ampicillin resistance cassette, a pBR322 origin of replication and a multiple cloning site (Figure S1 (map), Figure S2 (sequence) and Materials and Methods). Based on the organization of the plasmids developed by Wang *et. al.*, we then generated the *E. coli-C. trachomatis* shuttle plasmid, p2TK2-SW2, by introducing the *C. trachomatis* SW2 plasmid into the *Bam*HI restriction site of p2TK2 (Figure 1 (map), Figure S3 (sequence) and Materials and Methods).

**Figure 2:**
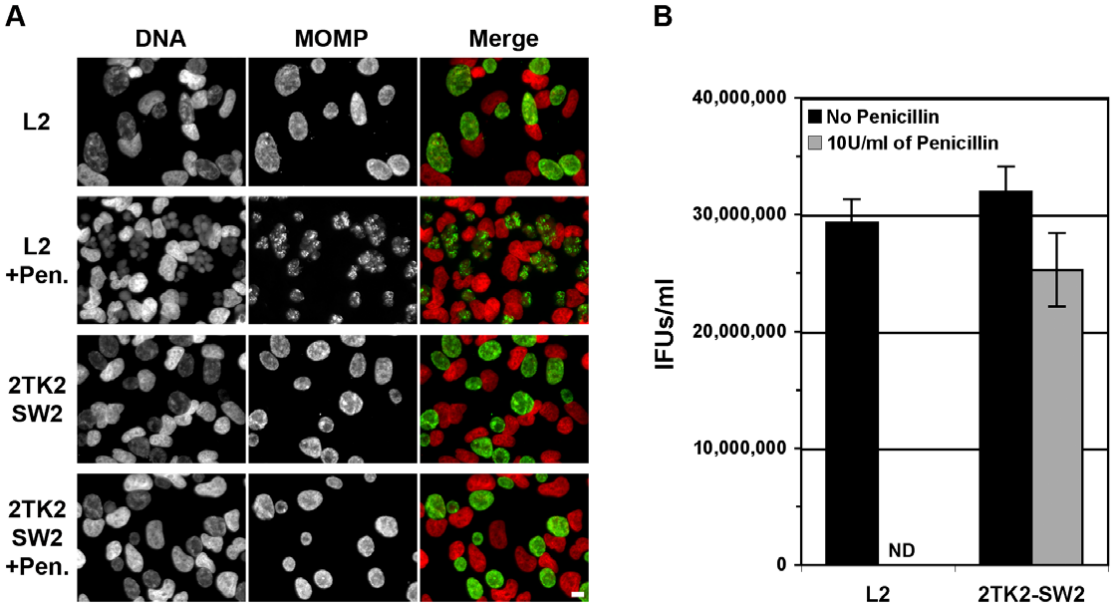
Growth characteristics of ***C. trachomatis*** L2 and the transformed strain harboring p2TK2--SW2. A. HeLa cells were infected with the parental strain, *C. trachomatis* L2, or the transformed strain harboring p2TK2--SW2 for 24 h in the absence (L2 and 2TK2SW2) or presence of penicillin (L2+Pen and 2TK2SW2+Pen). Fixed samples were stained with the DNA dye Hoechst (DNA, left panels) and antibodies against *Chlamydia* Major Outer Membrane Protein (MOMP) (MOMP, middle panels) were used to visualize the bacteria. The merge images are shown on the right (Red: DNA, Green: MOMP). Scale bar: 10 µm. B. HeLa cells were infected with the parental strain, *C. trachomatis* L2, or the transformed strain harboring p2TK2--SW2 in the absence (Black bars, No Penicillin) or in the presence of penicillin (Grey bars, 10 U/ml Penicillin). The number of infectious particles (IFUs) recovered 48 h post infection was determined. ND: None Detected.

The p2TK2-SW2 cloning vector was then introduced into *C. trachomatis* by incubating bacteria, DNA and HeLa cells in CaCl_2_/Tris Buffer as described by Wang *et. al.* ([37], Materials and Methods). Wild type inclusions were observed after two rounds of penicillin selection. After a third round of selection, the majority of the HeLa cells displayed large wild type inclusions. After clonal isolation of the transformants, we compared the growth characteristics of the parental and transformed strains (Figure 2). HeLa cells were infected with the parental strain, *C. trachomatis* L2, or the transformed strain harboring p2TK2-SW2 for 24 h in the absence or presence of penicillin. Fixed samples were stained with the DNA dye Hoechst and antibodies against *Chlamydia* Major Outer Membrane Protein (MOMP) were used to visualize the bacteria. As shown in Figure 2A, in the absence of penicillin the parental strain developed normal inclusion filled with wild type bacteria, whereas large aberrant bacteria were observed in the presence of penicillin. On the contrary, the transformed strain harboring p2TK2SW2, displayed inclusions harboring wild type bacteria both in the absence and in the presence of penicillin, confirming that the p2TK2--SW2 plasmid confers *C. trachomatis* resistance to penicillin.

We also compared the production of infectious particles of the parental and transformed strain harboring p2TK2--SW2 (Figure 2B). For this purpose, HeLa cells were infected with the parental strain, *C. trachomatis* L2, or the transformed strain harboring p2TK2-SW2 in the absence or presence of penicillin and the number of infectious particles recovered 48 h post infection was determined. In agreement with the immunofluorescence data, the parental strain was only able to produce infectious progeny in the absence of penicillin (Figure 2B, L2, black bar), whereas the transformed strain harboring p2TK2SW2 produced infectious particle both in the absence and in the presence of penicillin (Figure 2B, 2TK2SW2, black and grey bars). Importantly, equal amounts of progeny were recovered from both strains, indicating that the presence of the plasmid did not affect the production of infectious particles.

Altogether our results indicate that a *C. trachomatis* transformed strain harboring the p2TK2-SW2 cloning vector displayed resistance to penicillin and produced similar amount of infectious *C. trachomatis* to the parental strain.

### *C. trachomatis* transformants expressing RSGFP, mCherry or CFP from the *incD* gene promoter

**Figure 3:**
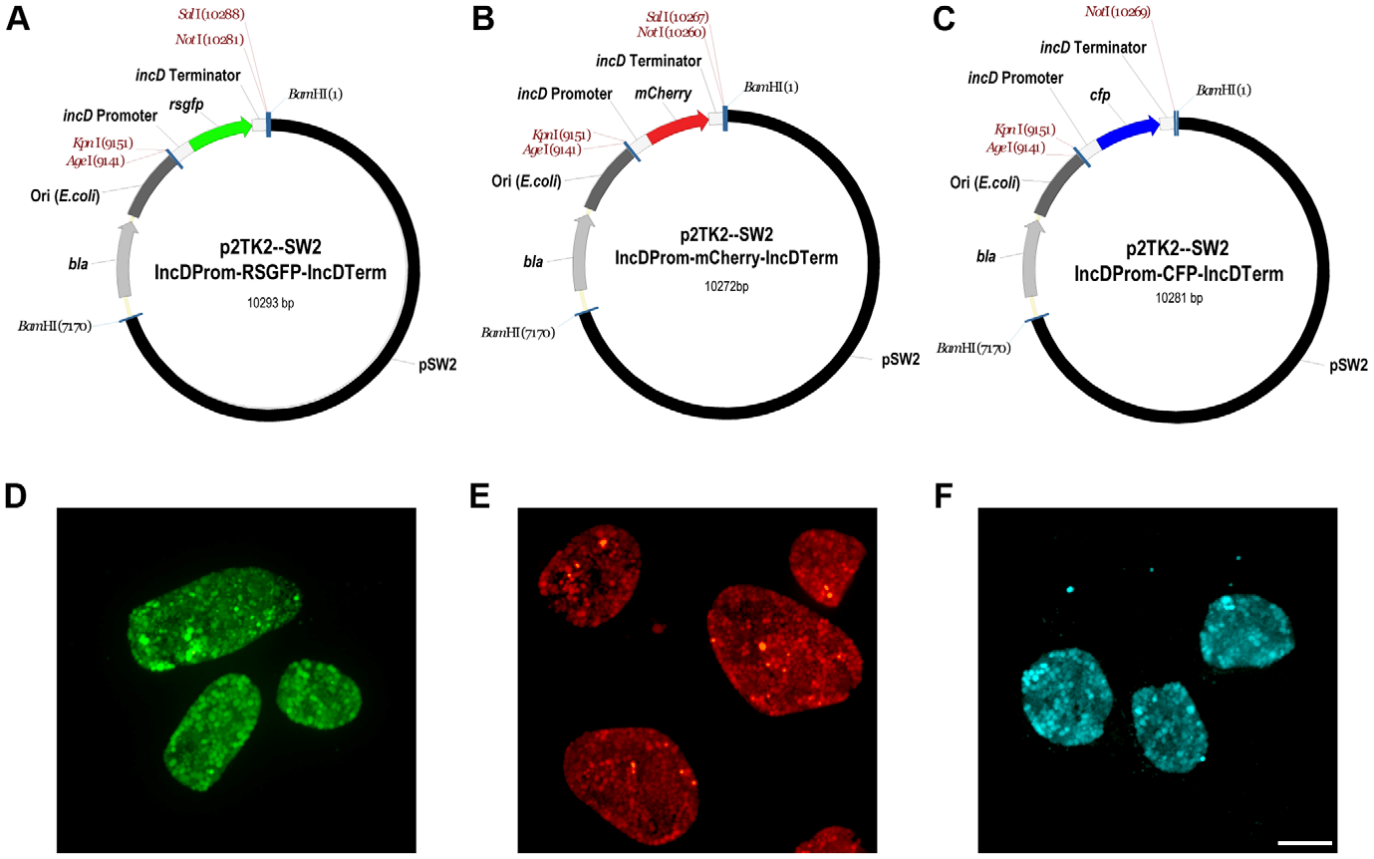
*C. trachomatis* transformants expressing RSGFP, mCherry or CFP from the ***incD*** **gene promoter**. A–C Maps of the p2TK-SW2 derivatives allowing expression of RSGFP (A), mCherry (B) or CFP (C) from the *incDEFG* operon promoter. The pSW2 plasmid is shown in black, the *E.coli* origin of replication in dark grey and the ampicillin resistance cassette (*bla*) in light grey. The *rsgfp* (green), *mCherry* (red) and *cfp* (blue) ORFs are flanked by the *incDEFG* operon promoter and terminator (light grey). Unique restriction sites are shown in red. D–F. Immunofluorescence images of *C. trachomatis* inclusions harboring bacteria expressing RSGFP (D), mCherry (E) or CFP (F) from the *incDEFG* operon promoter. Scale Bar: 10 µm.

We next generated versatile vectors allowing for expression of fluorescent proteins under the control of *C. trachomatis* regulatory elements. We chose the promoter and terminator regions of the *incDEFG* operon because it belongs to the class of *Chlamydia* genes that are expressed early and throughout the developmental cycle [41]. By overlapping PCR, we created a *Kpn*I/*Not*I DNA fragment where the *gfp* ORF was flanked by the promoter and terminator regions of the *incDEFG* operon. The *E. coli-C. trachomatis* shuttle plasmid, p2TK2-SW2 IncDProm-RSGFP-IncDTerm, was then generated by ligation of the IncDProm-RSGFP-IncDTerm DNA fragment into the *Kpn*I and *Not*I restriction sites of p2TK2-SW2 (Figure 3A (map), Figure S4 (sequence) and Materials and Methods).

We also tested whether additional fluorescent proteins, mCherry and the Cyan Fluorescent Protein (CFP), could be expressed in *C. trachomatis*. For this purpose, we created *Kpn*I/*Not*I DNA fragments where the *mCherry* or *cfp* [42] ORFs were flanked by the promoter and terminator regions of the *incDEFG* operon. The *E. coli-C. trachomatis* shuttle plasmids, p2TK2-SW2 IncDProm-mCherry-IncDTerm and p2TK2-SW2 IncDProm-CFP-IncDTerm were respectively obtained by ligation of the IncDProm-mCherry-IncDTerm or IncDProm-CFP-IncDTerm DNA fragments into the *Kpn*I and *Not*I restriction sites of p2TK2-SW2 (Figure 3B–3C (map), Figure S5–S6 (sequence) and Materials and Methods).

The p2TK2-SW2 IncDProm-RSGFP-IncDTerm, p2TK2-SW2 IncDProm-mCherry-IncDTerm and p2TK2-SW2 IncDProm-CFP-IncDTerm plasmids were then introduced into *C. trachomatis* as described by Wang *et. al.* ([37], Materials and Methods). Wild type inclusions were observed after two rounds of penicillin selection. After a third round of selection, the majority of the HeLa cells displayed large wild type inclusions. In addition, *C. trachomatis* transformed with the plasmids listed above, respectively developed GFP-, mCherry- and CFP-positive inclusions (Figure 3D, 3E and 3F). The analysis of plasmid and genomic DNA from the parental and transformed strains confirmed the episomal status of the transformed plasmids and the integrity of the chromosomal *incDEFG* locus of the GFP-, mCherry- and CFP-expressing *C. trachomatis* strains. In addition, we confirmed that the pL2 plasmid had been exchanged for the transformed plasmid (Figure S7).

Altogether, these results indicated that different fluorescent proteins could be expressed in *C. trachomatis* without apparent impairment of the developmental cycle. Moreover successful expression of the *gfp*, *mCherry* or *cfp* transgenes from the *incDEFG* promoter was achieved indicating that *C. trachomatis* transcriptional regulatory elements can be used to drive the expression of transgenes from the transformed plasmids.

### *C. trachomatis* strains expressing RSGFP, mCherry or CFP from the *incD* promoter replicate as well as the parental strain

We next compared the growth characteristics of the transformed strains expressing GFP, mCherry or CFP, to the parental strain and the strain harboring an empty plasmid.

**Figure 4:**
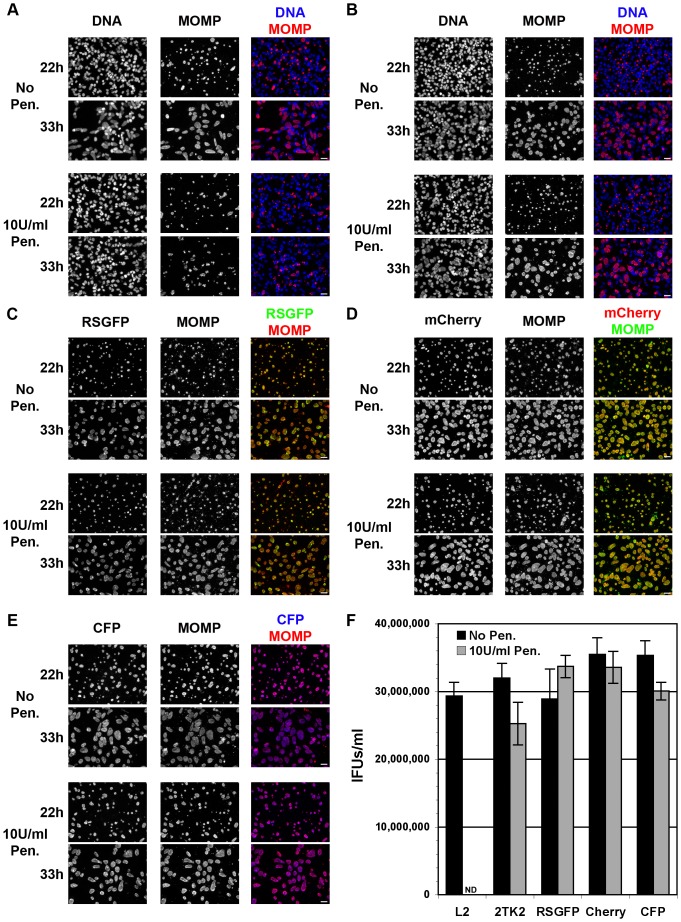
Growth characteristics of ***C. trachomatis*** L2 and the transformed strain expressing RSGFP, mCherry or CFP from the *incD* gene promoter. A–E HeLa cells were infected with the parental strain, *C. trachomatis* L2 (**A**), or the transformed strain harboring p2TK2-SW2 (**B**), p2TK2-SW2 IncDProm-RSGFP-IncDTerm (**C**), p2TK2-SW2 IncDProm-mCherry-IncDTerm (**D**) or p2TK2-SW2 IncDProm-CFP-IncDTerm (E) in the absence (No Pen.) or presence (10 U/ml Pen.) of penicillin. Samples were fixed 22 h and 33 h post infection and stained with the DNA dye Hoechst (**A**–**B**) (DNA, left panels) and antibodies against *Chlamydia* Major Outer Membrane Protein (MOMP) (**A**–**E**) (MOMP, middle panels) to visualize the inclusions. GFP (**C**), mCherry (**D**) and CFP (**E**) images are shown on the left panels. The merge images are shown on the right. Scale Bar: 25 µm. F. HeLa cells were infected with the parental strain, *C. trachomatis* L2 (L2), or the transformed strains harboring p2TK2-SW2 (2TK2), p2TK2-SW2 IncDProm-RSGFP-IncDTerm (RSGFP), p2TK2-SW2 IncDProm-mCherry-IncDTerm (Cherry) or p2TK2-SW2 IncDProm-CFP-IncDTerm (CFP) in the absence (Black bars, No Pen.) or in the presence of penicillin (Grey bars, 10 U/ml Pen.). The number of infectious particles (IFUs) recovered 48 h post infection was determined. ND: Not Detected.

HeLa cells were infected with the parental strain, *C. trachomatis* L2 (Figure 4A), or the transformed strains harboring p2TK2-SW2 (Figure 4B), p2TK2-SW2 IncDProm-RSGFP-IncDTerm (Figure 4C), p2TK2-SW2 IncDProm-mCherry-IncDTerm (Figure 4D) or p2TK2-SW2 IncDProm-CFP-IncDTerm (Figure 4E). Infections were performed in the absence (No Pen.) or in the presence (10 U/ml Pen.) of penicillin. Infected cells were fixed 22 h and 33 h post infection and stained with antibodies against *Chlamydia* Major Outer Membrane Protein (MOMP) to visualize the inclusions. As shown in Figure 4, 22 h post infection all five strains had developed wild type inclusions of equal size in the absence of penicillin (22 h, No Pen.). As the developmental cycle progressed, the size of the inclusion increased equally between the strains (33 h, No Pen.). Except for the parental strain, which displayed aberrant inclusions in the presence of penicillin, all transformed strains were able to develop wild type inclusions in the presence of penicillin (22 h 10 U/ml Pen. and 33 h 10 U/ml Pen.). The MOMP staining showed that even in the absence of penicillin, 100% of the inclusions were GFP- (Figure 4C), mCherry- (Figure 4D) or CFP-positive (Figure 4E), indicating homogeneity of the respective strains and stability of the plasmids for at least one round of infection cycle.

To determine whether the transformed plasmids were stable over time in the absence of penicillin, the mCherry strain was cultured for 20 developmental cycles and the GFP and CFP strains for 10 developmental cycles, in the absence of selection. To determine the homogeneity of the population after 20–10 developmental cycles, the cells were infected with the passaged strains at a MOI of 0.5 and 0.1 for 24 h (not shown). The fixed samples were stained with an antibody against MOMP. For all three strains, 100% of the MOMP-positive inclusions were fluorescent, demonstrating the stability of the plasmid over time, even in the absence of selection.

We next tested whether GFP-, mCherry- or CFP-expressing *C. trachomatis* produced the same amounts of infectious particles as the parental strain or the strain harboring an empty plasmid. For this purpose, HeLa cells were infected with the parental strain, *C. trachomatis* L2 (L2), or the transformed strains harboring p2TK2-SW2 (2TK2), p2TK2-SW2 IncDProm-RSGFP-IncDTerm (RSGFP), p2TK2-SW2 IncDProm-mCherry-IncDTerm (mCherry) or p2TK2-SW2 IncDProm-CFP-IncDTerm (CFP) in the absence (No Pen.) or presence (10 U/ml Pen.) of penicillin. The number of infectious particles recovered 48 h post infection was determined. As shown in Figure 4F, the parental strain only produced infectious particles in the absence of penicillin (L2, black bar), whereas the transformed strains produced similar amounts of infectious particles in the absence or presence of the antibiotic (2TK2, RSGFP, mCherry and CFP, black and grey bars respectively). In addition, the number of infectious particles recovered from the transformed strains in the absence or presence of penicillin was similar to the parental strain in the absence of penicillin.

Altogether, these results confirmed that *C. trachomatis* strains expressing GFP, mCherry or CFP developed inclusions of similar size to the parental strain or a strain harboring an empty plasmid, but also produced similar amount of infectious particles, indicating that expression of fluorescent proteins by *C. trachomatis* did not impair the progression and completion of the developmental cycle.

### *C. trachomatis* strains expressing RSGFP, mCherry or CFP from the *incD* promoter recapitulate major features of the *C. trachomatis* developmental cycle

**Figure 5:**
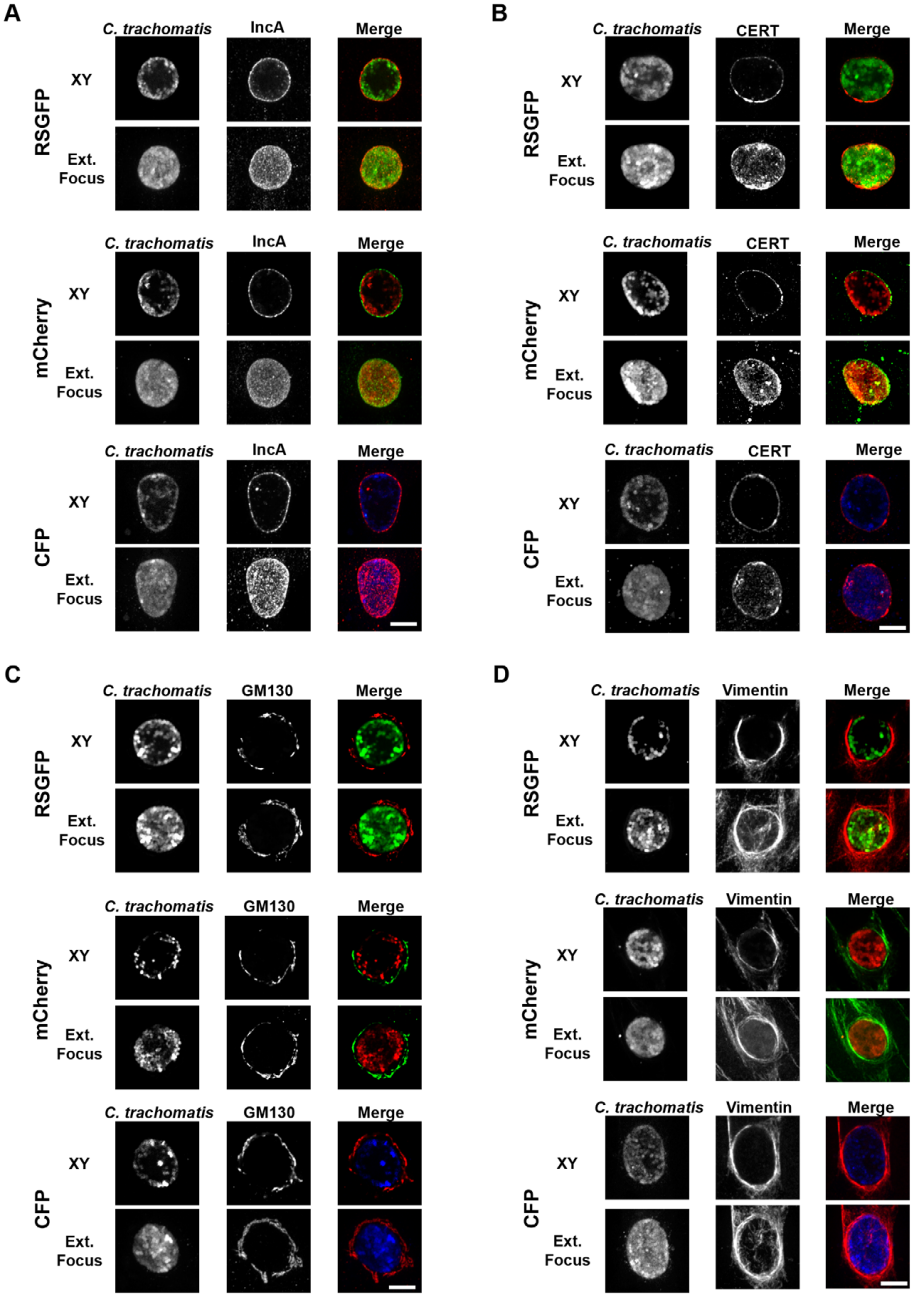
***C. trachomatis*** strains expressing RSGFP, mCherry or CFP from the ***incD*** gene promoter recapitulate major features observed during *C. trachomatis* developmental cycle. A–D. HeLa cells were infected with *C. trachomatis* transformed strains harboring p2TK2-SW2 IncDProm-RSGFP-IncDTerm (RSGFP), p2TK2-SW2 IncDProm-mCherry-IncDTerm (mCherry) or p2TK2-SW2 IncDProm-CFP-IncDTerm (CFP) for 24 h. Samples were fixed and stained with antibodies against IncA (A), CERT (B), GM130 (C) and Vimentin (D). Images were acquired using a spinning disc confocal microscope. For each strain and each marker, an XY view (Top panels, XY) and an extended focus view (Bottom Panels, Ext.Focus) are shown. Scale bar: 10 µm.

We next tested whether *C. trachomatis* strains expressing GFP, mCherry or CFP were able to recapitulate major features observed during the *C. trachomatis* developmental cycle. For this purpose, HeLa cells infected with fluorescent *C. trachomatis* strains were fixed 24 h post infection and we analyzed the localization/recruitment of bacterial and host proteins, as well as eukaryotic organelles, to the inclusion membrane by immunofluorescence (Figure 5).

Using anti-IncA antibodies, we tested whether IncA, a type III effector that belongs to a set of *C. trachomatis* proteins that are inserted into the inclusion membrane (Inc proteins) [43]–[45], was present in the inclusion membrane (Figure 5A). As a host factor associated with the inclusion membrane, we tested for the presence of the Ceramide transfer protein, CERT ([14], [15]), using anti-CERT antibodies (Figure 5B). Finally, Golgi ministacks and intermediate filaments, two eukaryotic structures known to surround *C. trachomatis* inclusion [13], [46], were respectively detected using antibodies against GM130 (Figure 5C) and Vimentin (Figure 5D).

As depicted in the extended focus views presented in Figure 5, IncA decorated inclusions harboring GFP-, mCherry- and CFP-expressing *C. trachomatis* and the XY views confirmed that the IncA signal was restricted to the inclusion membrane. Similarly, CERT was associated with the inclusion membrane where it clustered in small patches as previously observed [14], [15]. In addition, Golgi ministacks were observed all around and in the vicinity of the inclusion as described by Heuer *et. al.* [13] and the inclusions were also surrounded by a Vimentin cage as described by Kumar *et. al.* [11].

Altogether these observations indicate that fluorescent *C. trachomatis* strains recapitulated important features of the *C. trachomatis* developmental cycle such as translocation and incorporation of type III effectors into the inclusions membrane, association of host proteins with the inclusion membrane and recruitment/manipulation of cellular organelles.

### Applications of fluorescent *C. trachomatis* strains to the study of the developmental cycle

One of the main applications of fluorescent *C. trachomatis* strains is the study of the developmental cycle using time-lapse video microscopy. It is possible to record mid to late stages of the *Chlamydia* developmental cycle by phase contrast or differential interference contrast (DIC) microscopy, because of the characteristic morphology of the inclusion [13], [39], [47], [48]. Alternatively, GFP-expressing cells, in which the *Chlamydia* inclusion appeared as a black hole, were used to study bacterial release [49]. However, imaging of the early stages of the *Chlamydia* developmental cycle is challenging, if not impossible.

**Figure 6:**
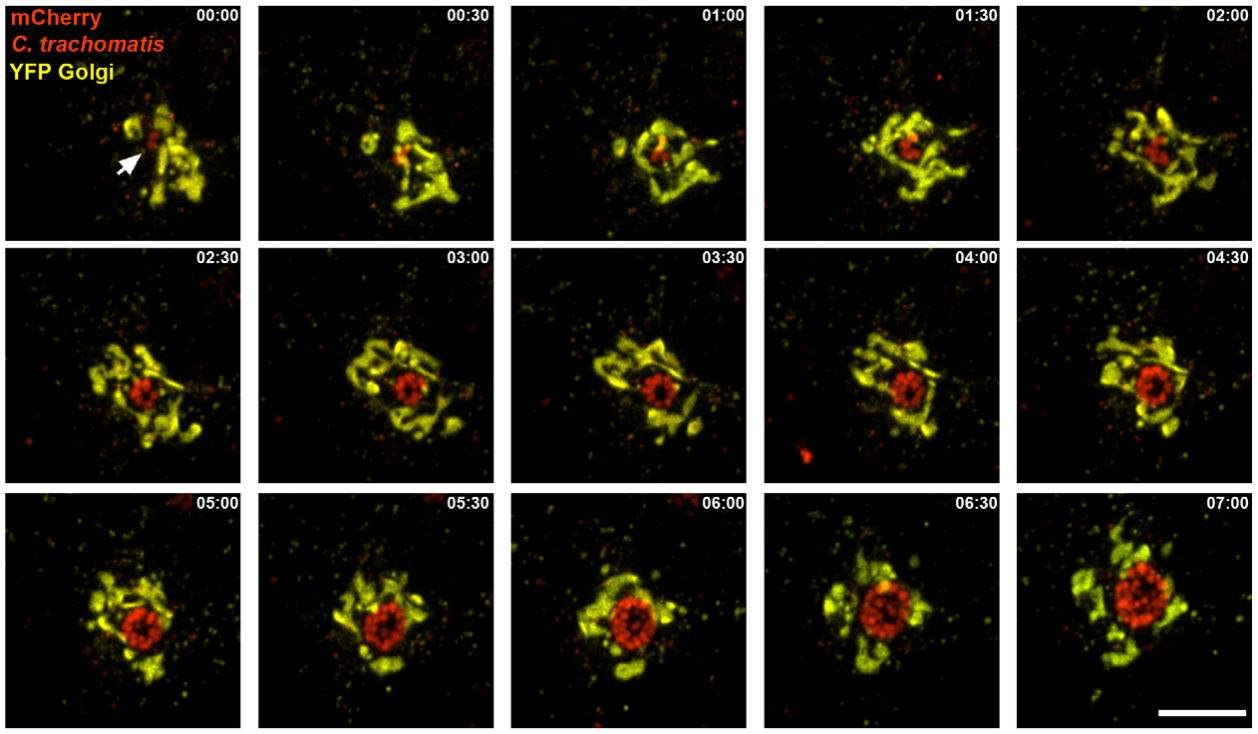
Time-lapse video microscopy of the association of *C. trachomatis* inclusion with the Golgi apparatus during the early stages of the developmental cycle. Selected merged frames from Video S1 acquired every 30 minutes by time-lapse video microscopy of HeLa cells transiently transfected with a YFP-Golgi construct (yellow) and infected with *C. trachomatis* expressing mCherry under the control of the *incD* promoter (red). The first frame corresponds to 10 h post infection. The time (hours: minutes) is indicated in the upper right corner of each frame. Scale Bar: 10 µm.

Using the *C. trachomatis* strain that expressed mCherry under the control of the *incD* promoter, we were able to image bacterial replication and the early association of the inclusion with the Golgi apparatus (Figure 6 and Video S1). For this purpose, HeLa cells were transfected with a YFP-Golgi construct (Clontech) 18 h prior infection with a *C. trachomatis* strain that expressed mCherry under the control of the *incD* promoter. The cells were monitored by spinning-disc confocal microscopy by acquiring series of z-stacks every 30 minutes. As previously described [13], we observed that the inclusion was ultimately surrounded by Golgi ministacks, but the use of a fluorescent *C. trachomatis* strain allowed us to monitor the early steps of this process (Figure 6 and Video S1). We were able to visualize the expansion of a nascent inclusion that initially contained 2 RBs (Figure 6, 00∶00, white arrow). As the infectious cycle progressed, we visualized bacterial replication leading to an increased number of bacteria that progressively lined up against the inclusion membrane giving the typical ring like pattern (Figure 6, 03∶30). The nascent inclusion was first closely apposed to the Golgi apparatus (Figure 6, 00∶00, white arrow) but very quickly nested itself inside the Golgi apparatus (Figure 6, 01∶00) where it continued to expand, surrounded by the Golgi. This phenomenon was observed in most of the cells that we imaged and suggested that very early on, inclusions containing 2–4 bacteria are already surrounded by the Golgi apparatus and that fragmentation of the Golgi into ministacks happens as the inclusions expand. This is different from previous observations that used phase contrast microscopy to monitor the inclusion and showed Golgi fragmentation after the inclusion had reached a certain size [13]. Both mechanisms are however not incompatible, as Golgi fragmentation might occur more or less early during the developmental cycle. In addition, our data do not contradict the fact that Golgi fragmentation is more apparent at later time point. Overall, our results demonstrate that the fluorescent *C. trachomatis* strains will be useful to investigate early interaction of the inclusion with host organelles.

Finally, we have used the fluorescent *C. trachomatis* strains to monitore inclusion fusion. *Chlamydia* inclusions have been shown to undergo homotypic fusion, a process that is inhibited at low temperature and depends on the inclusion protein IncA [44], [50]–[53]. We monitored this process by co-infection of HeLa cells with GFP- and mCherry-expressing *C. trachomatis* strains.

**Figure 7:**
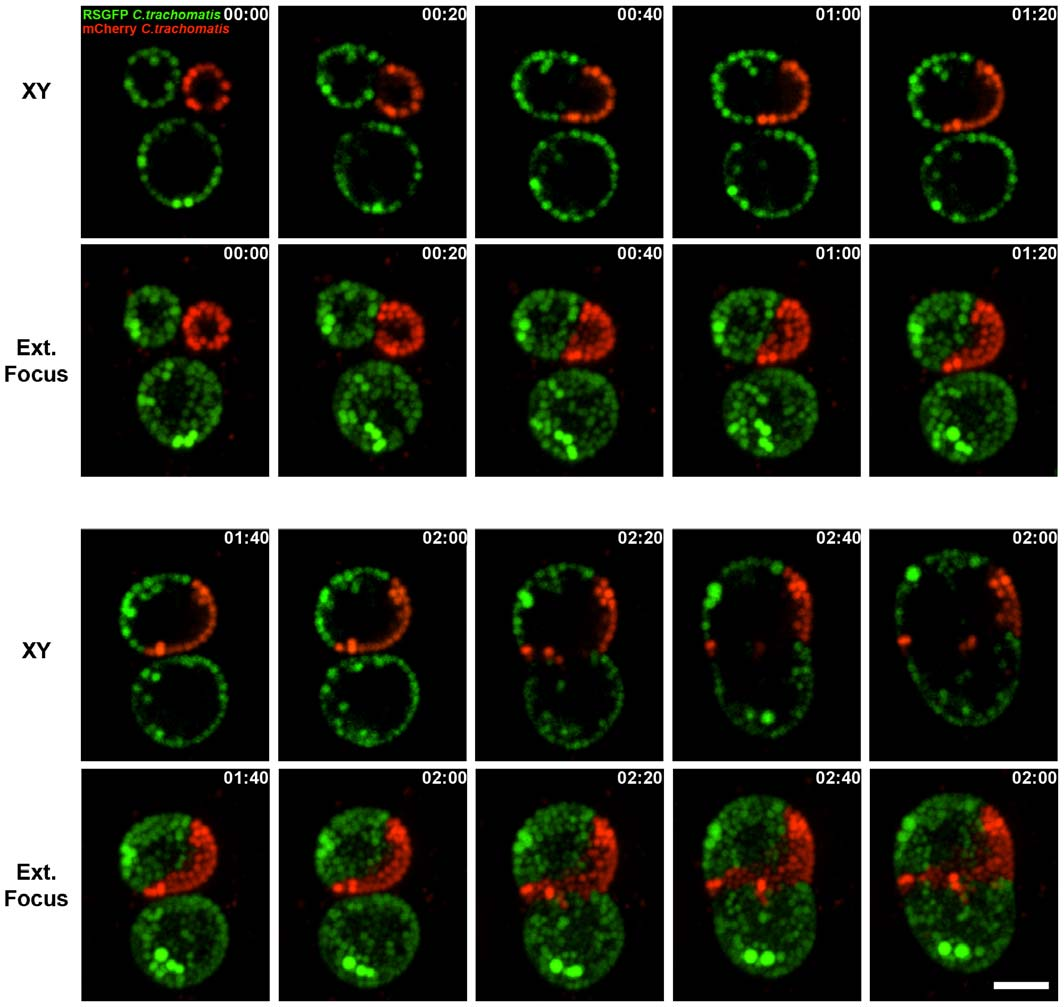
Time-lapse video microscopy of *C. trachomatis* inclusion fusion. Selected merged frames from Video S2 acquired every 20 minutes by time-lapse video microscopy of HeLa cells co-infected with *C. trachomatis* strains expressing GFP (green) or mCherry (red) under the control of the *incD* promoter. The first frame corresponds to 24 h post infection. For each time point, an XY view (Top panels, XY) and an extended focus view (Bottom Panels, Ext.Focus) are shown. The time (hours: minutes) is indicated in the upper right corner of each frame. Scale Bar: 6 µm.

In a first set of experiments, we monitored inclusion fusion 24 h post infection (Figure 7 and Video S2). For this purpose, HeLa cells were co-infected with *C. trachomatis* strains expressing GFP and mCherry under the control of the *incD* promoter. The multiplicity of infection was such that 1 to 3 inclusions per cell were observed 24 h post infection. Cells that displayed at least 2 inclusions were then monitored by spinning-disc confocal microscopy 24 h post infection, by acquiring series of z-stacks every 20 minutes. As shown in Figure 7 and Video S2, we were able to monitor inclusion fusion in a cell harboring 2 GFP-positive and 1 mCherry-positive inclusion at the beginning of the acquisition (Figure 7, 00∶00, Video S2). As the infection progressed, one of the GFP-positive inclusions fuses with the mCherry inclusion (Figure 7, 00∶20, Video S2) leading to an inclusion in which GFP-positive bacteria are lined up on one side of the inclusion membrane and mCherry positive bacteria on the other side. As time progressed, the bi-colored inclusion eventually fused with the second GFP positive inclusion (Figure 7, 02∶20, Video S2).

In a second set of experiments, HeLa cells were co-infected with *C. trachomatis* strains expressing GFP and mCherry under the control of the *incD* promoter, at a high multiplicity of infection (∼10 *C. trachomatis*/HeLa cells). Growth of the bacteria was monitored 10 h post infection by spinning-disc confocal microscopy, by acquiring series of z-stacks every 30 minutes. As shown in Video S3, at the beginning of the acquisition the cells were co-infected with several GFP and mCherry expressing bacteria. As the developmental cycle progressed, both bacterial replication and inclusion fusion occurred. Half way through the movie, the cells harbored several inclusions. Some contained only GFP- or mCherry-positive bacteria making it difficult to determine whether inclusion fusion had occurred. While other inclusions clearly resulted from fusion since they harbored both GFP- and mCherry-positive bacteria. By the end of the movie, each cell harbored a single inclusion containing GFP- and mCherry-positive bacteria and resulting from the fusion of the multiple inclusions observed at earlier time points.

While the fusion of medium to large size inclusion could have been monitored by phase contrast microscopy, the use of fluorescent bacteria allowed visualization of fusion of small inclusions containing few bacteria (Video S3). In addition, the use of bacteria of different colors allowed visualizing the segregation of each strain inside the inclusion (Video S2 and S3). Finally, co-infection with *C. trachomatis* strains expressing different fluorescent proteins would be a useful tool to unambiguously determine if inclusion fusion occurred by assaying for the presence of bacteria of each color at the end of the developmental cycle.

## Conclusions

We have generated versatile vectors for conducting genetic experiments in *C. trachomatis*. *C. trachomatis* strains expressing fluorescent proteins from *C. trachomatis* regulatory element will be useful to study temporal gene expression as well as the early developmental stages of the developmental cycle by time lapse-video microscopy. In addition, our cloning vector will be a valuable tool for mutant complementation as well as expression of wild type, mutated or tagged proteins in *C. trachomatis*.

## Supporting Information

Figure S1 **A minimal plasmid for replication in *E. coli.* Map of p2TK2.** The *E.coli* origin of replication and the ampicillin resistance cassette are respectively shown in dark and light grey. Unique restriction sites of the multiple cloning site are shown in red.
(TIF)

Figure S2 **A minimal plasmid for replication in *E. coli.* p2TK2 Vector Sequence.**
(DOC)

Figure S3 **A cloning vector for *C. trachomatis.* p2TK2-SW2 Vector Sequence.**
(DOC)

Figure S4 **p2TK2-SW2 IncDProm-RSGFP-IncDTerm Vector Sequence.**
(DOC)

Figure S5 **p2TK2-SW2 IncDProm-mCherry-IncDTerm Vector Sequence.**
(DOC)

Figure S6 **p2TK2-SW2 IncDProm-CFP-IncDTerm Vector Sequence.**
(DOC)

Figure S7 **Episomal status of the transformed plasmids and integrity of the *incDEFG* locus in the transformed strains.** A. Plasmids were isolated from the parental strain (L2) and from the transformed strains (mCh, GFP and CFP). The plasmids were digested with the *BamH*I restriction enzyme. As expected the pL2 plasmid was linearized, leading to a 7,505 bp DNA fragment. Digestion of the plasmids from the transformed strained generated a 7,176 bp DNA fragment, corresponding to the pSW2 plasmid, and to a ∼3,100 bp DNA fragment, corresponding to the p2TK2 plasmid and the insert containing the fluorescent protein gene flanked by the *incD* promoter and terminator. These results demonstrate the episomal status of the plasmid and the exchange of the pL2 plasmid for the transformed plasmid. B. DNA was isolated from the parental strain (L2) and from the transformed strains (mCh, GFP and CFP). The procedure led to isolation of both genomic and plasmid DNA. The DNA served as a template in PCR reactions using primers annealing upstream and downstream from the *incD* promoter and terminator respectively, either in the transformed plasmid (P) or the *C. trachomatis* chromosome (C). Please see the Material and Methods section and Table S1 for the name and sequence of the primers. As expected, when DNA from the mCherry or CFP strains was used as template (mCh and CFP), primers annealing to p2TK2-SW2 IncDProm-mCherry-IncDTerm or p2TK2-SW2 IncDProm-CFP-IncDTerm led to the amplification of a 1,300 bp DNA fragments corresponding to the mCherry or cfp gene flanked by the *incD* promoter and terminator. When DNA from the RSGFP strains was used as template (GFP), primers annealing to p2TK2-SW2 IncDProm-RSGFP-IncDTerm, led to the amplification of a 1,200 bp DNA fragment, corresponding to rsgfp gene flanked by the *incD* promoter and terminator. No DNA product was obtained when DNA from the parental strain was used as template (L2). Primers annealing to the chromosome led to the amplification of a 2,200 bp DNA fragment corresponding to the *incDEFG* operon, when DNA from the parental or the transformed strains was used as template. These results indicate that the *incDEFG* operon locus is not altered in the transformed strains. C. Southern blot using DNA (genomic and plasmid) isolated from the parental strain (L2) and from the transformed strains (mCh, GFP and CFP). The DNA was digested with the *BamH*I restriction enzyme and analyzed by Southern blot using probes corresponding to the p2TK2 plasmid or the pSW2 plasmid. As expected, a ∼3,100 bp DNA fragment, corresponding to the p2TK2 plasmid and the insert containing the fluorescent protein gene flanked by the *incD* promoter and terminator was only detected in samples from the transformed strains with the p2TK2 probe. The SW2 probe led to the detection of a 7,505 bp DNA fragment corresponding to the pL2 plasmid in the parental strain DNA sample and to the detection of a 7,176 bp DNA fragment corresponding to the pSW2 plasmid in the transformed strains DNA samples. These results further confirm the episomal status of the transformed plasmids and demonstrate the exchange of the pL2 plasmid for the transformed plasmid.
(TIF)

Table S1 Primers used in this study.
(DOC)

Video S1 **Time-lapse video microscopy of the association of *C. trachomatis* inclusion with the Golgi apparatus during the early stages of the developmental cycle.** Video S1 shows the association of *C. trachomatis* inclusion with the Golgi apparatus that occurs during *C. trachomatis* infection. HeLa cells were transfected with a YFP-Golgi construct (Yellow) 18 h prior infection with a *C. trachomatis* strain that expressed mCherry under the control of the *incD* promoter (red). 10 h post infection, a YFP-Golgi expressing cell was monitored by spinning disc confocal microscopy. A z-stack of 56 images covering 14 µm was acquired every 30 minutes. The QuickTime video was generated using Improvision Volocity. The time (hours: minutes: seconds) is indicated in the upper right corner. The video is played at 2 frames/s. Scale Bar: 3.5 µm.
(MOV)

Video S2 **Time-lapse video microscopy of *C. trachomatis* inclusion fusion 24 h post infection.** Video S2 shows the fusion of inclusions harboring GFP or mCherry expressing *C. trachomatis*. HeLa cells were co-infected with a *C. trachomatis* strain that expressed GFP (green) or mCherry (red) under the control of the *incD* promoter. 24 h post infection, a cell containing 2 GFP- and 1 mCherry-positive inclusion was monitored by spinning disc confocal microscopy. A z-stack of 56 images covering 14 µm was acquired every 20 minutes. The QuickTime video was generated using Improvision Volocity. The time (hours: minutes:seconds) is indicated in the upper right corner. The video is played at 2 frames/s. Scale Bar: 8 µm.
(MOV)

Video S3 **Time-lapse video microscopy of *C. trachomatis* inclusion fusion**. Video S3 shows the fusion of inclusion harboring GFP- or mCherry-expressing *C. trachomatis*. HeLa cells were co-infected with a *C. trachomatis* strain that expressed GFP (green) or mCherry (red) under the control of the *incD* promoter. 10 h post infection, cells containing several GFP- and mCherry-positive bacteria were monitored by spinning disc confocal microscopy. A z-stack of 56 images covering 14 µm was acquired every 30 minutes. The QuickTime video was generated using Improvision Volocity. The time (hours: minutes: seconds) is indicated in the upper right corner. The video is played at 2 frames/s. Scale Bar: 19 µm.
(MOV)
