# Supplementary figures and images for: A C. trachomatis Cloning Vector and the Generation of C. trachomatis Strains Expressing Fluorescent Proteins under the Control of a C. trachomatis Promoter
Source: PLoS One. 2013 Feb 18;8(2):e57090. doi: 10.1371/journal.pone.0057090 (PMC3575495; doi:10.1371/journal.pone.0057090)

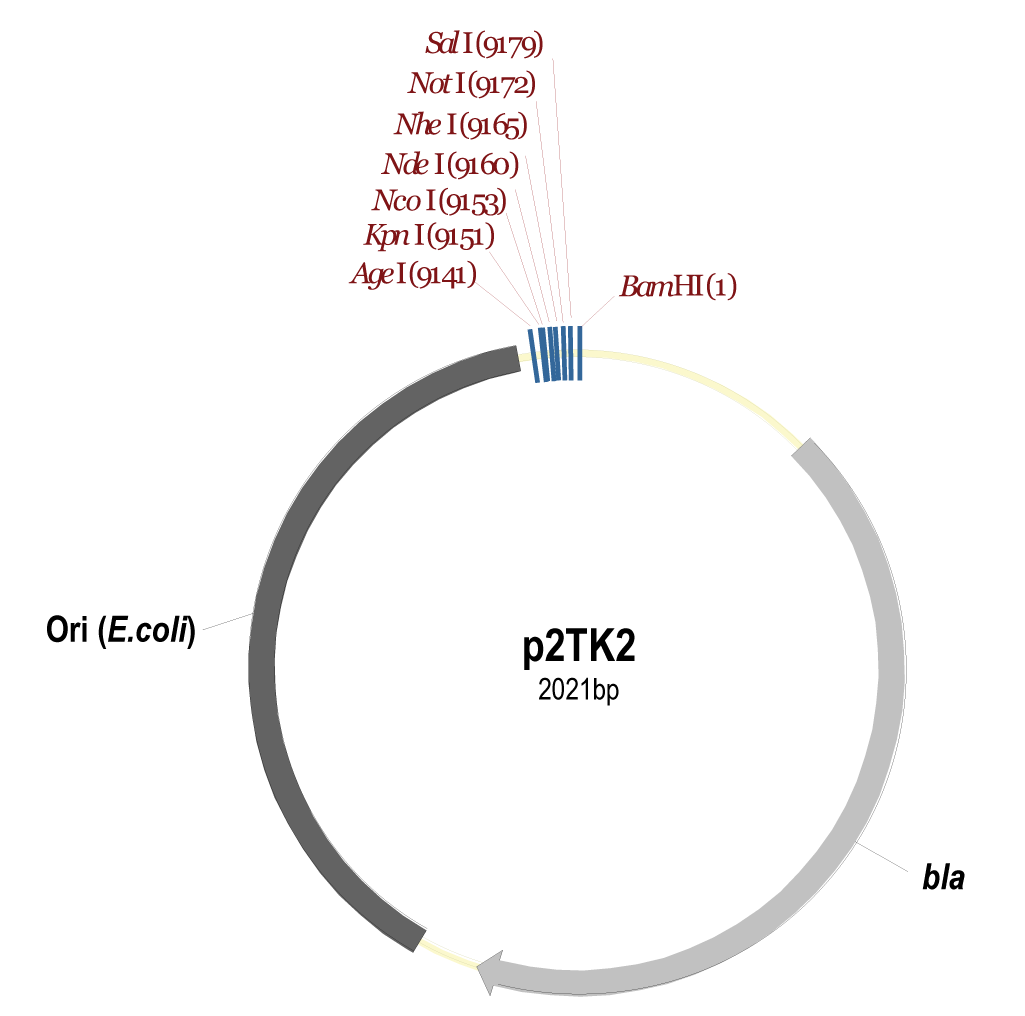

Supplement: Figure S1 — A minimal plasmid for replication in E. coli. Map of p2TK2. The E.coli origin of replication and the ampicillin resistance cassette are respectively shown in dark and light grey. Unique restriction sites of the multiple cloning site are shown in red. (TIF) [file pone.0057090.s001.tif]

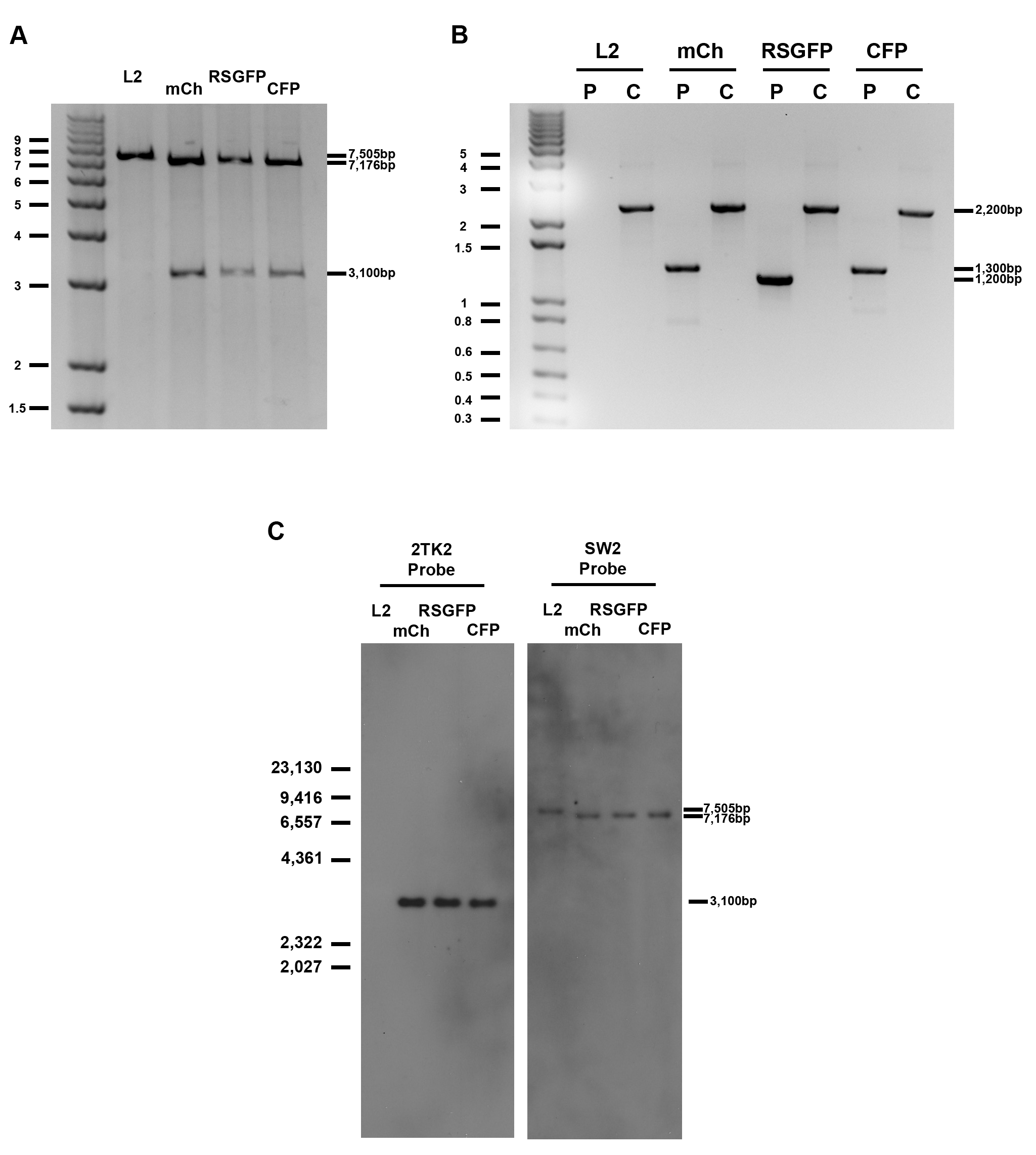

Supplement: Figure S7 — Episomal status of the transformed plasmids and integrity of the incDEFG locus in the transformed strains. A. Plasmids were isolated from the parental strain (L2) and from the transformed strains (mCh, GFP and CFP). The plasmids were digested with the BamHI restriction enzyme. As expected the pL2 plasmid was linearized, leading to a 7,505 bp DNA fragment. Digestion of the plasmids from the transformed strained generated a 7,176 bp DNA fragment, corresponding to the pSW2 plasmid, and to a ∼3,100 bp DNA fragment, corresponding to the p2TK2 plasmid and the insert containing the fluorescent protein gene flanked by the incD promoter and terminator. These results demonstrate the episomal status of the plasmid and the exchange of the pL2 plasmid for the transformed plasmid. B. DNA was isolated from the parental strain (L2) and from the transformed strains (mCh, GFP and CFP). The procedure led to isolation of both genomic and plasmid DNA. The DNA served as a template in PCR reactions using primers annealing upstream and downstream from the incD promoter and terminator respectively, either in the transformed plasmid (P) or the C. trachomatis chromosome (C). Please see the Material and Methods section and Table S1 for the name and sequence of the primers. As expected, when DNA from the mCherry or CFP strains was used as template (mCh and CFP), primers annealing to p2TK2-SW2 IncDProm-mCherry-IncDTerm or p2TK2-SW2 IncDProm-CFP-IncDTerm led to the amplification of a 1,300 bp DNA fragments corresponding to the mCherry or cfp gene flanked by the incD promoter and terminator. When DNA from the RSGFP strains was used as template (GFP), primers annealing to p2TK2-SW2 IncDProm-RSGFP-IncDTerm, led to the amplification of a 1,200 bp DNA fragment, corresponding to rsgfp gene flanked by the incD promoter and terminator. No DNA product was obtained when DNA from the parental strain was used as template (L2). Primers annealing to the chromosome led to the amplification of a 2,200 bp DNA fra [file pone.0057090.s007.tif]
