# Supplementary material for: A C. trachomatis Cloning Vector and the Generation of C. trachomatis Strains Expressing Fluorescent Proteins under the Control of a C. trachomatis Promoter
Source: PLoS One. 2013 Feb 18;8(2):e57090. doi: 10.1371/journal.pone.0057090 (PMC3575495; doi:10.1371/journal.pone.0057090)
Supplement: Figure S6 — p2TK2-SW2 IncDProm-CFP-IncDTerm Vector Sequence. (DOC) [file pone.0057090.s006.doc]

**Plasmid p2TK2--SW2 IncDPromoter-CFP-IncDTerminator features**

pSW2: bases 6-7167

Ampicillin resistance gene: 7416-8276

*E.coli* origin of replication: bases 8341-9124

*incD* Promoter: bases 9152-9379

*cfp* ORF: bases 9380-10099

*incD* Terminator: bases 10100-10266

Unique Restriction Sites: *Age*I (9141), *Kpn*I (9151) and *Not*I (10269)

**Plasmid p2TK2--SW2 IncDPromoter-CFP-IncDTerminator Sequence**

>p2TK2--SW2 IncDPromoter-CFP-IncDTerminator (direct) 10281bp

GATCCCTTGTACAATCAATTTACCGATTAAATAGTCTCTATAATTCACTATCCGGAGCGCTTCAAAAAAA

ACTGTCCATTCCTGCTTAGAAATCGATTCTGTTTTGATTTTGTCTCGGATTTTAAAAAATGTAGTGTTTC

CAAAATCTTTCAATGGAATAGCGGGTTTAATATATCCCTTGGTCAATCTATACAAAAACTTTGTGAAAGA

TATGTAGCATGCCGCTCTAGCCTGTTTAGATGCCTCTGAAACAACTTTTCCATTAAAAACATCTAGAGAC

TTGATTTTAAACAAAGATTCGCTGTGGTCAAGAGAAATAGCCTTTATCAAGGTTTCCGATAAATCCAGAA

TCTCTAAAGAAACAAGAAAGTTAATCCCAGACGCATAATTTTTTCTAGTTAGATAAGATAAAGTAGATAA

CCAAATTTCCGACGCGTCCCCAAAAGTTAAAAACAATCTACTTTTATGGAAAGCCATCGAGCCCATTTTC

TTAACCAAAGCTATTCAAAATCGGAGCTCTAAGATTTTAAGAAATTTTTTAACAAAAGTCCATTATGACC

AAGTCTACCACCAAGAGTTGCAAAGTCTACCACCAAGAGTTGCAAAGTCTACCACCAAGAGTTGCAAAGT

CTACCACCAAGAGTTGCAAATCTCTCTCGTGAAATCAAATCCCTAAATATATATATATAATAGATATATA

TATATGAGCTGACGGAGGATCAGCTCTTTTGCTTAAAAAGTTCAAAAAGCTGTTGTAGAAGATTTTCGTT

ATAGGAGGACAAAGAAACTCCGGAACACATGATGCGAAGTATCTCTATTAAGAAATCAGATAATTGGCGA

TTCTTCTCTGAATCAGACTTATCTATCGTTTCTCTAACGTCTTTGTTTCTAGATGAAGGAAGAAATTGAT

CCAACACCCTTATCGCCGATGAGTTCGACATTCCACATACTTTCCCTATCACATCGACCTTGGTTTTTAA

ATCGCCTTTTCTAGCGGCCAAAATATATGCGGATTTATAGGGGATCGATTGAAACTCTTTTTGTAGAGTT

TGGTTGGGGAGGTTTATAAAAAGCTCGTAATATGCAAGAGCATTGTAAGCAGAAGACTTAGTTCTAAAAA

CTAACTCTATCCAAGATGAAAAAGTTGTTGAGGAGAAGTGATCCTTACTCAGGATTTTTCTAGCATTATA

GATTTTTTCTCCTAAAAGAAGTACGTGTTGCTTCTGTATGGATTTTATCTGACCAGTAAGCAGTTTTACC

GCTAGGATGTCTTCTTGATAAAATTCTTCATCCGAATAGTTTTGGGACTCTGATAAAAATAATCGATCCA

AACTCTGACTTTCCTCAGAATTCAAAGTTGCTGAGAATAGTTCAATGGAGGGAAGCGTCTTCTTAAAATC

TAGAGAAGCGGCAGTTTGATTTTTTTTAAAAAAGACATCCGCTTCTTTTTTTAGTTTGTTCACGTTGTCC

TCTGAGAGTAATCTCGTTCATATTCGATATGCAAAATATTTGCTATTTCATGCGTTAACTTCAGAATATC

TTCTGCGGCCCTAGAATTTGGATAGACATTAGCTACAGAATCTTCTTTAAGAAGAGAACGGCTGAGAGAA

ATATCTCGACGAATTTTTGTTGAAAAAAGCTTGTTTTTGTAAATAGACTCGATAATGTCTATATACATTT

GGTTAGTCGAGTTACGATCATCCCAAAAAGACAAAGCTATTCCAAGAATGTGTTCTTCTTCAGGTTTTCC

GACCGAACTTAAGAATTCACGTATCTTTTGTAACCCTAGAATAGAAAAAGGTTCTGGAGTTAAACAAGCA

ATTAATTTGTCTCCTGCAACAAAAGCTTCTTTCGTTAACCCTCCTAGGCTAGGTGGAGTGTCTATTATGC

AGATGTCATAAAAAGGAGCGCAGTACTCATTCAGAAATAACTTTAAGTTGTTACTAGGTCCTCTATGAAT

ATCCAATTCTCTAAACTGTTCGGATGAAAATGATGCAGGAATTAGGTCCACACTATCTTTTTTTGTTTCG

CAAATGATTGATTTTAAATCGTTTGATGTGTATACTATGTCGTGCAAGCCTTTTTGGTCACTTCTGACAC

TAGCCCCCAATCCAGAAGATAAATTGGATTGCGGGTCTAGGTCAGCAAGTAACACTTTTTTCCCTAAAAA

TTGGGCCAAGTTGCATCCCACGTTTAGAGAAAGTGTTGTTTTTCCAGTTCCTCCCTTAAAAGAGCAAAAA

ACTAAGGTGTGCAAATCAACTCCAACGTTAGAGTAAGTTGTCTATTCAGCCTTGGAAAACATGTCTTTTC

TAGACAAGATAAGCATAATCAAAGCCTTTTTTAGCTTTAAACTGTTATCCTCTAATTTTTCAAGAACAGG

AGAGTCTGGGAATAATCCTAAAGAGTTTTCTATTTGTTGAAGCAGTCCTAGAATTAGTGAGACACTTTTA

TGGTAGAGTTCTAAGGGAGAATTTAAGAAAGTTACTTTTTCCTTGTTTACTCGTATTTTTAGGTCTAATT

CGGGGAAATCTTTTTTCACATCTTTAACAATTTTAATAAAATCGTCCCTCACTTTTCTTTTATTTTGCAT

AACAAACCCCGTAATTCGAACTGTTTTCTCTAAATATAAAACCTATAAGAAAAATCCAATAAAAATTGTT

TAAGCGTTTGTTTGAGGTATTACCTCTAAAAAAGATACATTAGAAGTATTTGTTATTCCTAAAATATCAT

TGCCATTAGAAAGGGCATTAACCCATACCACACCGCTTTCTAAACCGCCTACACGTAATGAATACGTTGT

CGGAGTCAATCCTGTATTAGTAATACTGGTTCTTAGACTACATAAATTAGGAATGCCTGATGAGTATCCA

TAACTAATCGCGCAGGGCTTAGAATCACCTTCTCGTACCAAAGCTAGAACAACGCCGCCTTCCATTCTTG

ATGCAATAATATCTGCTGAGACTAAGAACATGCTCCCAGAGCTTTTGGGTGTGACTGTGAATTTTCCTAT

TTCAGTTCCTCCTAATAAAGTTTCAATGTTACTGGGAGTGAATAACCCGTTGCATTGAATTTTATTAGTG

ATTGGAAAGTTGTTAAAAGCTTTCAACAAACCTAGAGAAGGGTCTGTTTTGATTTTGTCTAAAATATCTT

GGACTGTACTATCAACAATAGTATCAGCAATTCCATCAAGAATTTGATCTCCCAACTTTTCTAGAATAAG

CTGGTAAGCTTTTTCCGCATCCAAACCAATTGTAATAGAAGCATTGGTTGATGAATTATTGGAGACTGTT

AAAGATATTCCATCAGAAGCTGTCATTTTGGCTGCGACAGGTGTTGATGTTGTCCCAAGGATTATTTGCT

GGTCCTTGAGCGGCTCTGTCATTTGCCCAACTTTGATATTATCAGCAAAGACGCAGTTTTCAGTGTTATA

CAAATAAAAACCAGAATTTCCCATTTTAAAACTCTTTTTTATTTTGAGCTTTAAATAAATTAGGTTTTTA

GTTTCAAGCTTGCTATTAATTAATAGATTCTTGTTCTAATTGTTCCATTTGTTCTTTAGATTTCTTAGTT

ATTTCTTCAAAGCGCTCTTTATTTAGATATAGAATTTCTTTTTTAGAGAGTTTAGAAGAATCCAGAAATT

CAATGCGTTTTCTTCTAGATAACCAGCCAAGCTTAATGGCGATTTCTATACATTTATCGATAGCTAACTC

GATTTTTTTCCAGTTCCTTGTACAGATGTACCGATTCATCCTTAAAATATATGCAAGACTTTTAACGTTA

ACGTTTAATAACAAGTTTTCTGGCCAAGAATTATCCTTAGTTAATTTTCGTCTCTTTTTCGCAGCTGCTG

TAATCACCCAGTCGATAAATGTGTAAGCATACTTTGATGCATTTGGGAAACGCATTTTTATTTCTTGGTA

TACATTTGCAGGCTTGATTACAAAGTAGGATTCTATTTGATCTACCAAGATAGGACATGGCTCTACAACG

AACCCTTTATGTTTCCGTGTAGGTGGTGAATTAAAAGGTGTTAAGTCTATATCTATATTTTCTTCGTCAG

TTAAACCTTCCCATCCTTCGTAAATCCTAATGATCGGAGAAAGAGTTTGGTAACGGTCTACTATTTGTGT

TCCATTAGTCCATCGAGTTCTAGTTGCCACTATTAAAAACGGTTGATGTCCTAAATGGTATAAGGCTTCT

AAAGCAGTTTCAGCTTCTTTTCCACTAAACTCATACTTATTTCTGGATGTTTTATACCGCTTAACTCCAT

AAGCCTCTAAGAATTCAGTTTTTGTAAAACGGATTCTTGGTATCCATCCTTCAAATTGAAAACTATTTGA

TTCTCTGGATAAAACAACCCCTTTTGTGTTCCCCTTGTAATTCGTTGCGGTCAGTAATCTTTGGATAGCT

GCTAATGCATGGTAATGAGATGAAAGAAAATCAAGACCTATAACTTCTACCATCCCATTTTGAGCCAATT

TGGGAGATATCTTAATAGATTGACCAGGTCTTCTTCCAAACTTCTGATTTTCAAGGTGGATAGGACTTTT

GATGAAGTGGCAGTTACTATAATTTACCATACTTTTTTAATAGCGGAGAATTTACTAATTTTTGGATCGA

AATGTAATACCGAAGAGAAAACCGATCCATGTCTATTTTTCCCAACAGTTATCTCACAATTAGAAGACGA

TTCCTTCCTATTGATAAACAAAATCACATCTGCGTCTTGCTCTATTTGACCGCTGTCTCGCAAATCTGAA

AGCATGGGAACTTTATTTGCTCTATCCTCAACTTTTCTAGATAGTTGGGATAAACAAACTATAGGAATGT

TTAGCTCTGAGGCTAAACCTCTTAAGGTTCTAGATATATCTGCTATTTCATTTTGACGATTTTCTCCAAC

CGATGAGTTGATCAACTGCAAGTAATCGATAAATATTACGTCTACTCGATCTTCTTTTCTCAGCAACCGG

ATCTGATTCGCGATTAAGTTAAGCTTATACTGACTATCACTGCAGATATAAAAATGTGATTCTCTAACCG

TTTCTCCAGCTTCTTCTACTCGGAATAATTCTTCTTTAGAGAGATCCCCTCTTTGTAATTTTTCACCAGA

TATTCCTGTTAAATTAGCAATAATCCGCTCAACAATTTGACCTGCGCTCATTTCTAGAGATAGGAAACCA

ACTCTACGCTGTTGAGTAACCGCAAGATTTATCGCCATGTCTATAGCTAAAGCTGTTTTCCCTATAGATG

GTCTAGCTGCTATAATCACGAAATTACCTTTAGCTAAGATAACTCCTTTATCATCAATATCCTTGTATCC

TGTTGGGAAGCCATCAAAGAAAGAATTTTGATTCTCAGAGAACGCTACTCGTCTTTTTTTTATACGAGCC

AGCACTCCAATTTCTGACTGTGAGAATATATCATAAATAGACCGGCCTCTAGCGCTGCGAATAGAAAAAG

TCTTTGCTATAGCACTATCAAGCCTTCCCTTTATACGCTCAAGCAATAGAAACGGAGATCTACGCAATGG

ATTTTCATTGTACTCATTAAACGAGCGGAAAATGAAATTACTCAAATTTTCTTCAGCGCTACACACGCTC

AAATCATCGAGGAAAACCGTATGAGAAACGGATCTAAGCTTGTCATTTGATAAAGCATCATGCAACATTA

ACCCGAGATACGATTTGTCCATATCTTTGATACGACGCCGCAAAAGCTCTTCCCAAGCCGAGTCTACAGT

TATAGGTAATCCATTGTCTTTTAAGTATTTAAATACTATGAATATGTTTTTATGATGAGAACACTTAAAC

TCATAATTAGCAAGCTGCCTCAGAATATACTCAGTAGAGTCTTCAAATATCAGAGCTTTACCTAACAACG

CATACTCGATATCTTGCATGCGATTTTCTATTTCGGAACGAGTTTTCATGTTTATATAAAAAAATACCGA

GCGTGCTATCCTGTTAACAACCTGTTTATATAAAAAAATACCGAGCGTGCTATCCTGTTAACAACCTGAT

TATTTCACTAATCAGGACATTTTACGGATAGGTTATATCACGAGGGATTTCATGGGTAAAGGGATTTTAT

CTTTGCAGCAAGAAATGTCGTTAGAATATAGTGAAAAGTCTTATCAGGAAGTTTTAAAAATTCGCCAAGA

ATCCTATTGGAAACGCATGAAAAGCTTCTCCTTATTCGAAGTTATTATGCATTGGACCGCATCACTCAAC

AAACATACTTGTAGATCATATCGAGGATCTTTTTTGTCTTTAGAAAAGATTGGTCTATTGTCCTTGGATA

TGAATCTGCAAGAGTTTTCCCTTTTAAATCATAATCTAATCCTAGATGCGATTAAAAAAGTTTCCTCTGC

CAAGACTTCTTGGACCGAAGGTACTAAACAAGTTCGAGCAGCAAGCTATATTTCCTTAACAAGATTCCTA

AACAGGATGACTCAAGGAATAGTCGCTATAGCGCAACCTTCTAAACAAGAAAATAGTCGAACATTTTTTA

AAACCAGGGAAATAGTAAAAACGGATGCGATGAACAGTTTGCAAACAGCATCCTTCCTAAAAGAGCTAAA

AAAAATCAATGCCCGGGATTGGTTGATCGCCCAGACAATGCTCCAAGGAGGTAAACGCTCCTCTGAAGTC

TTAAGCTTGGAGATTAGTCAGATTTGTTTCCAACAAGCTACCATTTCTTTCTCCCAGCTTAAGAACCGTC

AGACAGAAAAGAGGATTATTATAACTTATCCTCAGAAGTTTATGCACTTTCTACAAGAGTACATCGGTCA

ACGAAGAGGTTTTGTCTTCGTAACTCGCTCCGGAAAAATGGTGGGGTTAAGGCAAATCGCCCGCACGTTC

TCTCAAGCAGGACTACAAGCTGCAATCCCTTTTAAAATAACCCCGCACGTGCTTCGAGCAACCGCTGTGA

CGGAGTACAAACGCCTAGGGTGCTCAGACTCCGACATAATGAAGGTCACAGGACACGCAACCGCAAAGAT

GATATTTGCGTACGATAAATCTTCTCGAGAAGACAACGCTTCAAAGAAGCTGGCTCTAATATAGCCTAAA

GGTGTTTTTTCTGGCAACAGAATATGAATATAATTTTAATTATATCACAATATTGTGGGTGTTTGTACTA

GAGGAATTACCTCTTCCCCAGAACAAACGGATCCGACCCAGTCACGTAGCGATAGCGGAGTGTATAATTC

TTGAAGACGAAAGGGCCTCGTGATACGCCTATTTTTATAGGTTAATGTCATGATAATAATGGTTTCTTAG

ACGTCAGGTGGCACTTTTCGGGGAAATGTGCGCGGAACCCCTATTTGTTTATTTTTCTAAATACATTCAA

ATATGTATCCGCTCATGAGACAATAACCCTGATAAATGCTTCAATAATATTGAAAAAGGAAGAGTATGAG

TATTCAACATTTCCGTGTCGCCCTTATTCCCTTTTTTGCGGCATTTTGCCTTCCTGTTTTTGCTCACCCA

GAAACGCTGGTGAAAGTAAAAGATGCTGAAGATCAGTTGGGTGCACGAGTGGGTTACATCGAACTGGATC

TCAACAGCGGTAAGATCCTTGAGAGTTTTCGCCCCGAAGAACGTTTTCCAATGATGAGCACTTTTAAAGT

TCTGCTATGTGGCGCGGTATTATCCCGTGTTGACGCCGGGCAAGAGCAACTCGGTCGCCGCATACACTAT

TCTCAGAATGACTTGGTTGAGTACTCACCAGTCACAGAAAAGCATCTTACGGATGGCATGACAGTAAGAG

AATTATGCAGTGCTGCCATAACCATGAGTGATAACACTGCGGCCAACTTACTTCTGACAACGATCGGAGG

ACCGAAGGAGCTAACCGCTTTTTTGCACAACATGGGGGATCATGTAACTCGCCTTGATCGTTGGGAACCG

GAGCTGAATGAAGCCATACCAAACGACGAGCGTGACACCACGATGCCTGCAGCAATGGCAACAACGTTGC

GCAAACTATTAACTGGCGAACTACTTACTCTAGCTTCCCGGCAACAATTAATAGACTGGATGGAGGCGGA

TAAAGTTGCAGGACCACTTCTGCGCTCGGCCCTTCCGGCTGGCTGGTTTATTGCTGATAAATCTGGAGCC

GGTGAGCGTGGGTCTCGCGGTATCATTGCAGCACTGGGGCCAGATGGTAAGCCCTCCCGTATCGTAGTTA

TCTACACGACGGGGAGTCAGGCAACTATGGATGAACGAAATAGACAGATCGCTGAGATAGGTGCCTCACT

GATTAAGCATTGGTAACTGTCAGACCAAGTTTACTCATATATACTTTAGATTGATTTAAAACTTCATTTT

TAATTTAAAAGGATCTAGGTGAAGATCCTTTTTGATAATCTCATGACCAAAATCCCTTAACGTGAGTTTT

CGTTCCACTGAGCGTCAGACCCCGTAGAAAAGATCAAAGGATCTTCTTGAGATCCTTTTTTTCTGCGCGT

AATCTGCTGCTTGCAAACAAAAAAACCACCGCTACCAGCGGTGGTTTGTTTGCCGGATCAAGAGCTACCA

ACTCTTTTTCCGAAGGTAACTGGCTTCAGCAGAGCGCAGATACCAAATACTGTCCTTCTAGTGTAGCCGT

AGTTAGGCCACCACTTCAAGAACTCTGTAGCACCGCCTACATACCTCGCTCTGCTAATCCTGTTACCAGT

GGCTGCTGCCAGTGGCGATAAGTCGTGTCTTACCGGGTTGGACTCAAGACGATAGTTACCGGATAAGGCG

CAGCGGTCGGGCTGAACGGGGGGTTCGTGCACACAGCCCAGCTTGGAGCGAACGACCTACACCGAACTGA

GATACCTACAGCGTGAGCTATGAGAAAGCGCCACGCTTCCCGAAGGGAGAAAGGCGGACAGGTATCCGGT

AAGCGGCAGGGTCGGAACAGGAGAGCGCACGAGGGAGCTTCCAGGGGGAAACGCCTGGTATCTTTATAGT

CCTGTCGGGTTTCGCCACCTCTGACTTGAGCGTCGATTTTTGTGATGCTCGTCAGGGGGGCGGAGCCTAT

GGAAAAACGCCAGCAACGCGGCCTTTTTACGGTTCCTGGCCTTTTGCTGGCCTTTTGCTCACATGTTCTT

TCCTGCGTTATCCCCTGATTCTGTGGATAACCGTATTACACCGGTGGTACCAACGGAGCCTTCTAGCTAT

TTTGTAAATATTTTAACAATTTAGATTCTTCAAAGCTCAGCGAGGGCGTGAAGAATCTTGTTCAGGTGTA

TTTGAAAAAAGTTTGTTTTAAATAGTTTTTTTAGTTAAAATGGGTCCCTAAATAATTTAAATCCGGTAGT

TTTTGCGTCCGAAACATTGTTTTATAAGTGAGAAATGAGATCTGGCTAAAATCTGTCGAAGTGAGGTTTA

TGGCTAGCAAAGGAGAAGAACTTTTCACTGGAGTTGTCCCAATTCTTGTTGAATTAGATGGTGATGTTAA

TGGGCACAGATTTTCTGTCAGTGGAGAGGGTGAAGGTGATGCTACATACGGAAAGCTTACCCTTAAATTT

ATTTGCACTACTGGAAAACTACCTGTTCCATGGCCAACACTTGTCACTACTTTGACCTGGGGTGTTCAAT

GCTTTTCCCGTTATCCGGATCATATGAAACGGCATGACTTTTTCAAGAGTGCCATGCCCGAAGGTTATGT

ACAGGAACGCACTATATCTTTCAAAGATGACGGGAACTACAAGACGCGTGCTGAAGTCAAGTTTGAAGGT

GATACCCTTGTTAATCGTATCGAGTTAAAAGGTATTGATTTTAAAGAAGATGGAAACATTCTCGGACACA

AACTCGAGTACAACTATATCTCACACAATGTATACATCACGGCAGACAAACAAAAGAATGGAATCAAAGC

TCACTTCAAAATTCGCCACAACATTGAAGATGGAGGCGTTCAACTAGCAGACCATTATCAACAAAATACT

CCAATTGGCGATGGCCCTGTCCTTTTACCAGACAACCATTACCTGTCGACACAATCTGCCCTTTCGAAAG

ATCCCAAAGAAAAGCGTGACCACATGGTCCTTCTTGAGTTTGTAACTGCTGCTGGGATTACACGTGGCAT

GGATGAGCTCTACAAATAAGGATGACATGTGATTCGCGTAGGAAAAAGAGGAGGGAGACCTCCTCTTTTT

TTTTATTTTGTAGAGTTCCGTTACTATTGGCACCCTGTGTGCAGTTAGGATGAGTAGACTAGTTCTGCAG

CCTTTTACAGGGTGTTATGTTTTGCATTGCAAAAAGCTCCTAAGACGCGGCCGCGTCGACG
