# Supplementary material for: A C. trachomatis Cloning Vector and the Generation of C. trachomatis Strains Expressing Fluorescent Proteins under the Control of a C. trachomatis Promoter
Source: PLoS One. 2013 Feb 18;8(2):e57090. doi: 10.1371/journal.pone.0057090 (PMC3575495; doi:10.1371/journal.pone.0057090)
Supplement: Table S1 — Primers used in this study. (DOC) [file pone.0057090.s008.doc]

**Table S1: Primers used in this study.**

| **PRIMER NAME** | **PRIMER SEQUENCE** |
| --- | --- |
| Mod2TK2-5-Nde | catcatatggctagcgcggccgcgtcgacggatccGACCCAGTCACGTAGCGATAGC |
| Mod2TK2-3-Nde | catcatatgccatggggtaccaccggtGTAATACGGTTATCCACAGAATC |
| IncDProm&Orf-5-Kpn | GGTGGTACCaacggagccttctagctattttg |
| IncDTerm-3-Not | GCGGGCGGCCGCgtcttaggagctttttgcaatgc |
| RSGFP-START-5 | atctgtcgaagtgaggtttATGAGTAAAGGAGAAGCACTTTTC |
| RSGFP-START-3 | GAAAAGTGCTTCTCCTTTACTCATaaacctcacttcgacagat |
| RSGFP-STOP-5 | CAAGTCCGGACTCAGATCTTAAggatgacatgtgattcgcg |
| RSGFP-STOP-3 | cgcgaatcacatgtcatccTTAAGATCTGAGTCCGGACTTG |
| mCherry-START-5 | ctgtcgaagtgaggtttATGGTGAGCAAGGGCGAGGAGG |
| mCherry-START-3 | CCTCCTCGCCCTTGCTCACCATaaacctcacttcgacag |
| mCherry-STOP-5 | GGACGAGCTGTACAAGTAGggatgacatgtgattcgcg |
| mCherry-STOP-3 | cgcgaatcacatgtcatccCTACTTGTACAGCTCGTCC |
| CFP-START-5 | tctgtcgaagtgaggtttATGGCTAGCAAAGGAGAAGAAC |
| CFP-START-3 | GTTCTTCTCCTTTGCTAGCCATaaacctcacttcgacaga |
| CFP-STOP-5 | CATGGATGAGCTCTACAAATAAggatgacatgtgattcgcg |
| CFP-STOP-3 | cgcgaatcacatgtcatccTTATTTGTAGAGCTCATCCATG |
| SW2ProbeFwd | GTTTGTTCTGGGGAAGAGGTAATTCC |
| SW2ProbeRev | AAGGAGGTAAACGCTCCTCTGAAGTC |
| ChrUpIncDProm | gctatctcttgttgctccgag |
| ChrDwnIncDTerm | gtctgcatcaccctctac |
| PlasmUpIncDProm | GGATAACCGTATTACACCGG |
| PlasmDwnIncDTerm | CTTCCCCAGAACAAACGGATC |
| 2TK2Fwd | CGGTTCCTGGCCTTTTGCTGGCC |
| SW2Rev2 | TTCTAAGCAGGAATGGACAG |
